# Multifunctional nanocarriers for targeted drug delivery and diagnostic applications of lymph nodes metastasis: a review of recent trends and future perspectives

**DOI:** 10.1186/s12951-023-01990-4

**Published:** 2023-08-02

**Authors:** Huan-Rong Lan, You-Ni Zhang, Yue-Jun Han, Shi-Ya Yao, Meng-Xiang Yang, Xiao-Gang Xu, Xiao-Zhou Mou, Ke-Tao Jin

**Affiliations:** 1 Department of Surgical Oncology, Hangzhou Cancer Hospital, Hangzhou, 310002 Zhejiang Province China; 2 Department of Laboratory Medicine, Tiantai People’s Hospital, Taizhou, 317200 Zhejiang Province China; 3 Department of Colorectal Surgery, Affiliated Jinhua Hospital, Zhejiang University School of Medicine, Jinhua, 321000 Zhejiang Province China; 4 Center for Rehabilitation Medicine, Department of Ophthalmology, Zhejiang Provincial People’s Hospital (Affiliated People’s Hospital), Hangzhou Medical College, Hangzhou, 310014 Zhejiang Province China; 5 General Surgery, Cancer Center, Department of Hepatobiliary & Pancreatic Surgery and Minimally Invasive Surgery, Affiliated People’s Hospital, Zhejiang Provincial People’s Hospital, Hangzhou Medical College, Hangzhou, 310014 Zhejiang Province China; 6 Clinical Research Institute, Affiliated People’s Hospital, Zhejiang Provincial People’s Hospital, Hangzhou Medical College, Hangzhou, 310014 Zhejiang Province China

**Keywords:** Targeted drug delivery, Diagnostics, Lymph nodes metastasis, Nanoparticles, Tumour

## Abstract

Lymph node metastasis is a frequent occurrence in a variety of tumour forms and poses an enormous challenge to cancer treatment. This process is critical to the development of the disease and is frequently linked to a poor prognosis. Over 90% of cancerous cells move through lymph nodes, making them important entry routes for the spread of cancer cells. The prognosis of cancer patients is significantly impacted by lymph node metastases, which also affects treatment choices. Targeting lymph node metastases presents numerous difficulties for conventional medication delivery techniques. It is still very difficult to selectively target cancer cells in lymph nodes without risking injury to healthy organs and unforeseen consequences. Additionally, systemic delivery of drugs is hampered by the slow flow rate of lymphatic vessels. Chemotherapeutic medicines’ poor solubility and stability further reduce their effectiveness when taken orally. Additionally, the extracellular matrix that surrounds lymph node tumours is extensive, which makes it difficult for conventional pharmaceutical delivery systems to reach cancer cells. The development of nanocarriers for precise drug delivery to LNs has attracted a lot of interest to overcome these obstacles. Most solid tumours first spread through the lymphatic system, hence effective drug administration to these tissues is essential for better therapeutic results. Nanocarriers have several benefits, including the capacity to pass through barriers like blood-brain barriers and membranes to reach the lymphatic system. High medication dosages can be enclosed thanks to the physicochemical characteristics of nanocarriers, such as their higher surface-to-volume ratio. Additionally, ligands, antibodies, polymers, or biological molecules can be attached to nanocarrier surfaces to change their properties, allowing for the targeted delivery of lymph node epithelial cells. This use of nanocarriers for drug delivery maximizes on-target effects and related adverse effects while improving the effectiveness of medication delivery to target locations. More research and development in this field is needed to optimize nanocarrier design, increase targeting capabilities, and expand clinical applications for better cancer care.

## Introduction

Lymph node metastasis is a typical occurrence in the clinical context across a variety of tumour forms posing a significant challenge in cancer care. This nefarious process is closely linked to disease progression and is typically accompanied by a bleak prognosis [1]. Surprisingly, lymph nodes (LNs) appear to be major entrance routes for original cancer cell propagation; more than 90% of malignant cells pass through these sentinel organs [2]. Malignant tumour has different prognoses depending on whether lymph node metastases are present. As a result, the occurrence of metastases within LNs has a significant impact on the prognosis of cancer patients. Although the tumour, node, and metastasis (TNM) classification system gives primary consideration to metastatic LNs when deciding on staging, surgical excision of LNs proximal to the tumour is recognized as a frequent therapeutic practice [3].

The invasiveness of therapeutic options such as surgery, radiation, and chemotherapy, as well as the risk of major bad consequences, frequently render them impracticable and necessitate the discontinuation of ongoing therapies [4]. While radiation therapy and surgical intervention are routinely employed to remove lymph node metastases in the hopes of finding a cure, these procedures are fraught with complications such as lymphedema, prolonged hospital stays, and pain [5]. Surgical lymph node dissection has long been used as the gold standard for staging and removing metastatic LNs in cancer patients. During this invasive surgery, the affected LNs are removed and sent for pathological investigation to determine the extent of metastasis and inform future treatment options. Even though surgical lymph node dissection provides helpful staging information, it has a number of drawbacks. There is also the danger of severe morbidity and a lengthy recovery period, as well as complications such as infection and lymphedema. Furthermore, this technique may not always detect small or micro metastatic LNs correctly, resulting in under-staging [6, 7].

Furthermore, the survival of metastatic cells increases the likelihood of the disease returning [8]. Even though, systemic chemotherapy is effective in causing complete responses in lymph node metastasis, access to it is limited, and its concentration in metastatic LNs is short-lived, necessitating the use of high doses, increasing the risk of dose-related toxicities [9]. Sub-optimal drug doses, on the other hand, cause cancer recurrence and the formation of therapeutic resistance [10]. As a result, there is an urgent need for targeted delivery of chemotherapeutic medications to metastatic LNs, which offers a promising means of enhancing patient prognosis, quality of life, and treatment efficacy.

Nanoscale carriers have become a potential technique for the targeted administration of chemotherapeutic medicines to solid tumours following systemic treatment. This strategy aims to improve therapeutic efficacy while lowering the adverse pharmacological side effects that come with it [11]. Using systemically given nanocarriers, this approach holds great promise for targeting lymph node metastases regardless of their physical location. It has been difficult to amass nanocarriers selectively in metastatic LNs. Current nanoparticles (NPs) based approaches suffer from carrier building in healthy LNs due to their lymphotropic activity, which is dependent on tissue extravasation, absorption by lymphatic arteries or macrophages, and migration to draining LNs [12, 13]. Although this approach is effective in healthy nodes, the risk of side effects makes it less suitable for cytotoxic drug targeting. Furthermore, cancers with limited lymphatic outflow accumulated fewer NPs in their metastatic LNs [14]. As a result, intratympanic or local administration of carriers is the key element of current techniques for delivering nanocarriers to lymph node metastases [15]. These methods, however, are limited by unpredictable or heterogeneous drainage patterns [16], metastases that have spread to multiple nodes, drainage to multiple nodal basins, unknown primary sites [17], obstruction of lymphatic flow, or metastases that have spread to LNs outside of the surgical site [18]. Given these challenges, a systemic targeting method utilizing nanocarriers that may reach metastatic LNs via circulation has significant potential as an efficient mechanism for treating the entire scope of the metastatic condition.

The use of nanoscale carriers for the targeted delivery of chemotherapeutic medicines to solid tumour following systemic injection has shown promise in terms of boosting treatment efficacy while reducing associated side effects. Precision delivery to the tumour site is feasible by taking advantage of these carriers’ unique properties, such as their small size and surface modifications, which optimize therapeutic benefits while minimizing off-target side effects. The use of nanocarriers in cancer treatment is a novel strategy that promises improved results and improved patient well-being [19]. Targeting lymph node metastases with systemic nanocarrier injection provides a significant advantage since it allows for prospective therapy for metastatic LNs regardless of anatomical location. However, a persistent issue is the preferential accumulation of nanocarriers in lymph node metastases after systemic injection. Another impact of current NPs approaches, which rely on tissue extravasation, lymphatic channel or macrophage uptake, and migration to draining LNs, is the accumulation of NPs in healthy LNs [13].

By conducting a thorough review of the literature, this review highlights the developments and challenges associated with multifunctional nanocarriers in the treatment of lymph node metastases. This review aims to bridge knowledge gaps, provide comprehensive appraisals of the state of the subject, and identify future research directions. Finally, the inclusion of multifunctional nanocarriers into clinical praxis has the potential to transform the therapy of lymph node metastases, resulting in better patient outcomes and increased quality of life.

### Physiology and importance of lymph node

LNs, which are strategically situated between lymphatic channels, are an important aspect of the intricate lymphatic system found throughout the body [20]. As the primary sites for coordinating adaptive immune responses, these nodules play an important role in directing lymphocyte proliferation, replication, and activation [6]. Numerous studies have shown how important the lymphatic system is for the immune system to be able to recognize and react to diseases. Prior to haematological dissemination, the majority of solid tumours spread from the primary tumour site by infiltrating the local lymphatic tissue. This highlights the lymphatic system’s critical role in the early stages of cancer metastasis and highlights the system’s importance in the disease’s progression [21].

**Fig. 1:**
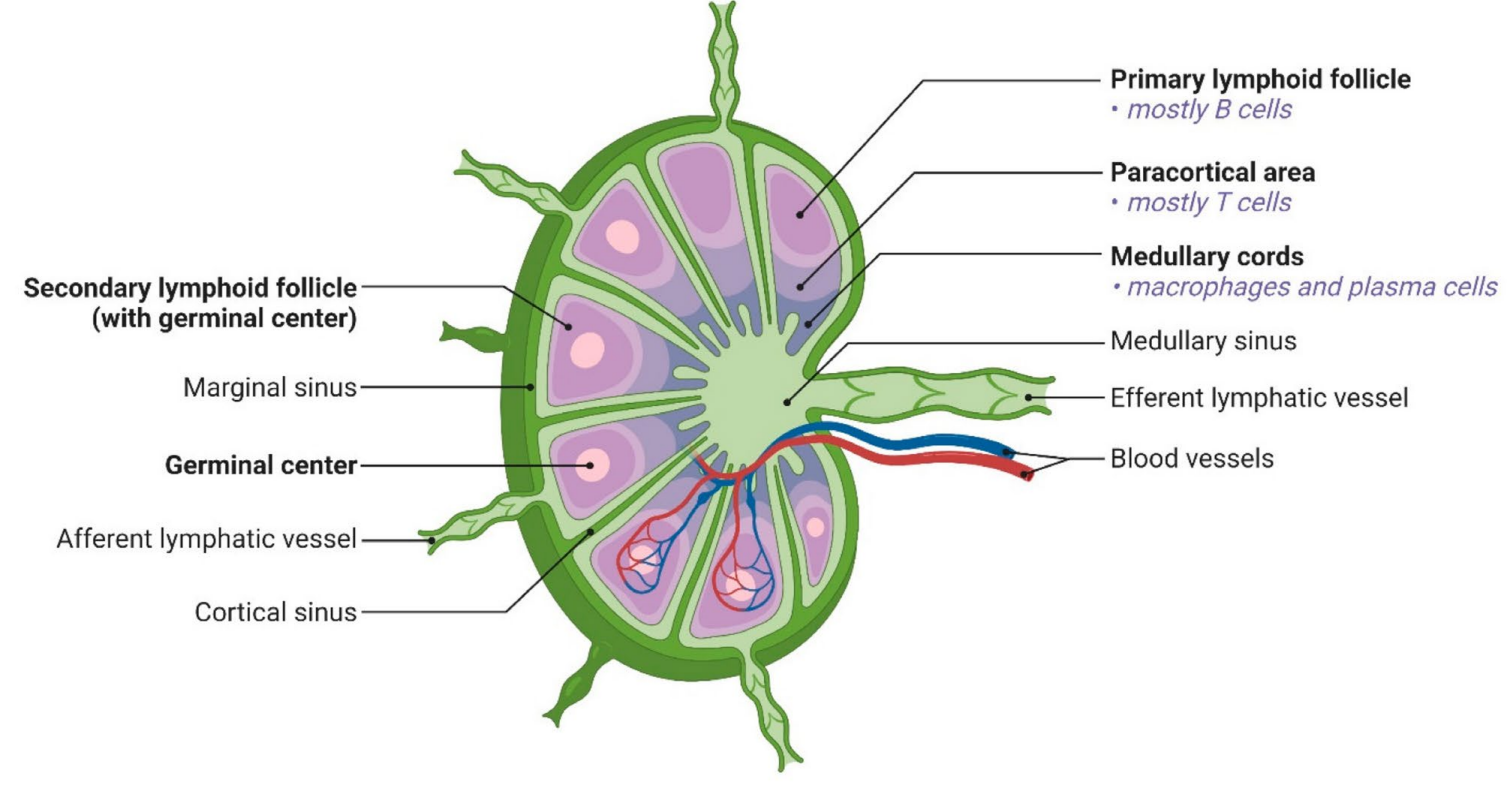
Unveiling the Complexities: Uncovering Lymph Node Structure and Mapping Lymphocyte Distribution. Adapted from [28] under the terms and conditions of the Creative Commons Attribution (CC BY) license (https://creativecommons.org/licenses/by/4.0/)

The kidney-shaped form of LNs allows them to accept lymphatic fluid from numerous afferent veins (Fig. 1). Later, the filtered lymph leaves the nodes through one or two efferent arteries. Each lymph node typically has an artery and a vein connected to it, which develops into a specialized vessel called a high endothelial venule (HEV). Trans-endothelial migration of circulating lymphocytes, aided by the interaction between T and B-cell surface receptors and the endothelial cells, occurs within the HEV and is a crucial mechanism [22].

Researchers have learned more about the greater influence lymphatic vessels exert on a spectrum of disorders, transcending earlier assumptions, because of our growing understanding of the complex physiological processes regulated by lymphatic regulation. It is increasingly clear that the lymphatic system plays a more active role in the pathogenesis and development of a variety of conditions, including inflammatory disorders, cancer metastasis, and tissue regeneration, in addition to its traditional roles in immune response and fluid homeostasis. LNs serve as sites for systemic defense in addition to initiating local defense mechanisms against infections and developing an immune response to fight cancer [23].

As a result of the expanding awareness of the critical role the lymphatic system, particularly LNs, plays in our immunological defense, scientists are focusing on delivering immune functional molecules, such as antigens and adjuvants, to LNs in order to trigger a powerful immune response. Tumour chemotherapy’s goal is to deliver accurate and tailored therapeutic doses to selected organs or cells. As a result, the development of novel nano-drug delivery systems aimed towards LNs has become a hot research area. This study’s purpose is to improve the efficacy and precision of medicine administration, which will advance therapeutic tactics in a range of sickness circumstances [24]. LNs are appealing therapeutic targets for addressing various unmet clinical needs. These challenges include eliminating B and T cell-derived malignant tumours, removing latent viral cell reservoirs, controlling sentinel lymph node metastases, improving vaccine efficacy, and encouraging immunological tolerance. The significance of LNs in various clinical situations has fueled research attempts to develop novel techniques to optimize their therapeutic potential [25]. Localization of LNs has been shown to significantly improve therapeutic outcomes in a range of therapeutic scenarios, including cancer and transplantation [11].

### Lymph node metastasis

**Fig. 2:**
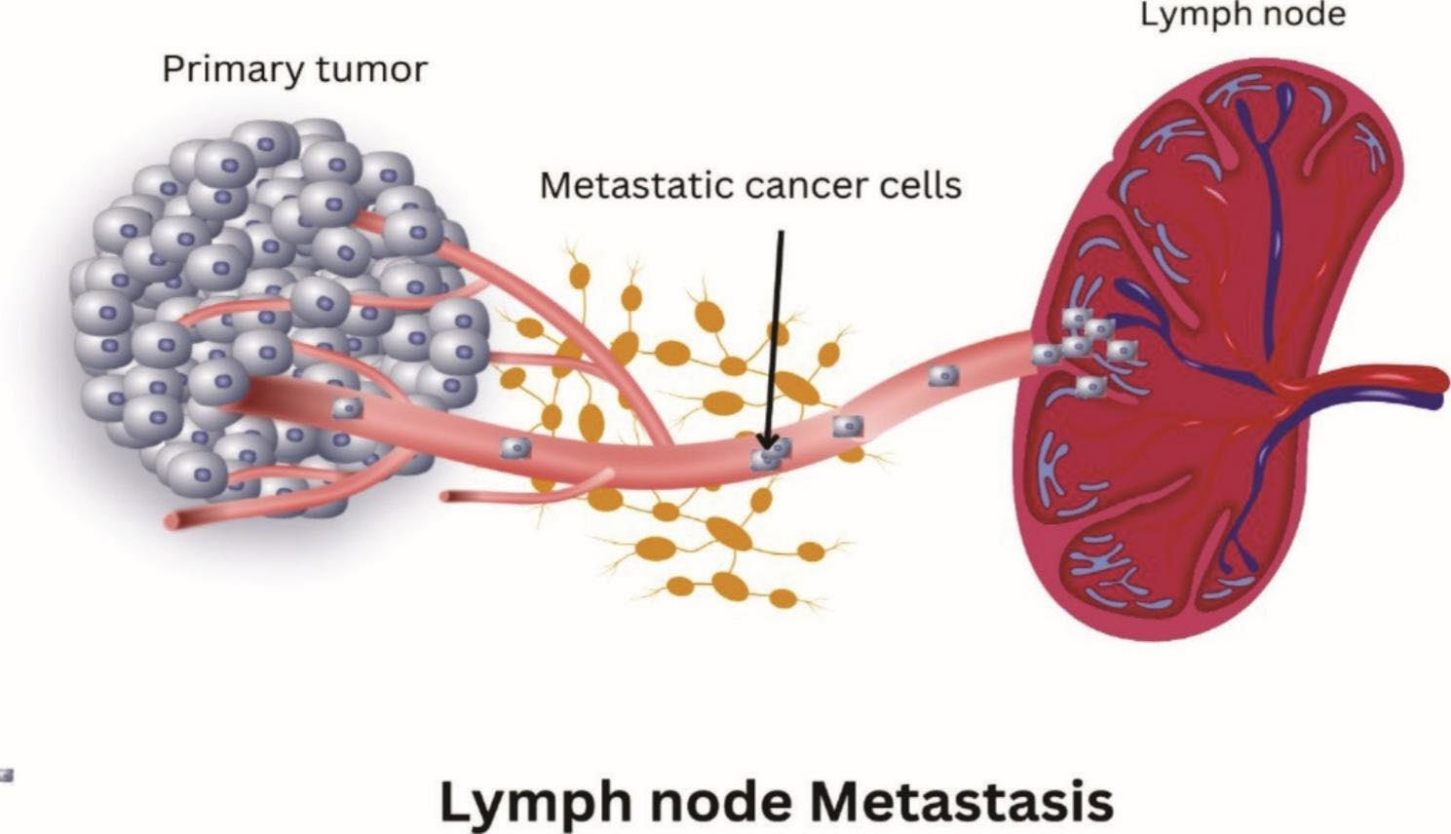
Illustration of metastasis of primary tumor into lymph node

Lymph node metastasis (LNM) is a well-known prognostic marker for a variety of solid tumours, including head and neck cancer (HNC). Lymph node metastasis is regarded as an important indicator of disease progression and has a significant impact on patients’ prognosis and treatment success [26]. It describes how cancer cells spread from a primary tumour to adjacent LNs via the lymphatic system (Fig. 2). This is common in many cancer types and is significant since it may indicate disease progression and have an impact on current treatments. Cancer cells that are present in LNs can reduce both the chance of distant metastasis and the overall survival rate [27]. A variety of factors influence lymph node metastasis, including tumour size, location, and type. An increased risk of lymph node metastasis is also associated with the presence of perineural invasion (PNI) and lymphovascular invasion (LVI). A few signaling pathways and genetic disorders have also been linked to the enhancement of LNM [27].

Treatment of lymph node metastases can be challenging for a variety of reasons. To begin, it can be difficult to detect early-stage metastases with existing imaging techniques. As a result, many patients may not begin receiving treatment until their sickness has progressed significantly. Second, traditional chemotherapy and radiation therapy may be ineffective in treating metastatic cancer due to medication resistance or toxicity concerns. Finally, certain people with severe disease or tumours in difficult-to-reach regions may not be candidates for surgical treatment [28].

### Challenges in drug delivery to lymph node

Traditional medicine administration systems have numerous limitations for lymph node metastases. One of the most significant disadvantages is the inability to selectively target cancer cells in LNs, which may have unexpected consequences and be hazardous to healthy organs. Furthermore, because of the low flow rate of lymphatic arteries, standard pharmaceutical delivery procedures typically rely on systemic administration, which may result in poor drug distribution. Furthermore, several chemotherapeutic medicines have low solubility and stability, which may limit their efficacy when administered conventionally. Finally, the extracellular matrix that surrounds lymph node tumours may prohibit traditional pharmaceutical delivery systems from accessing it, restricting their ability to target cancer cells [29]. It is difficult to deliver medications to LNs because of inadequate lymphatic outflow and immunological response. This implies that the lymphatic vessels’ cancer cells may not be effectively targeted by conventional drug delivery techniques. Drug delivery systems based on nanomaterials, however, have shown promise in addressing these difficulties [28].

Another challenge is that poor lymphatic drainage can be an issue, reducing the efficiency of drug delivery. A precise targeting strategy is required since either direct medicine delivery to LNs or lymph node immunization can result in immune tolerance. NPs can help to overcome these challenges and provide numerous benefits for lymph node medicine delivery. The NPs given in this manner can interact with the sinus’s phagocytic cells, but due to size constraints, they can only just barely infiltrate the T cell zone. As a result, when creating nanoparticles, the size and surface charge that may enter lymphatic capillaries, as well as strategies for infiltrating tumours in LNs, must be considered [2].

The difficulty of delivering drugs to specific tissues, such as the interstitium, lymphatics, and LNs, has resulted in the need for tailored drug administration. Because of their unique physiological constitution, these tissues are difficult for drugs to enter and remain in. For example, the extracellular matrix in connective tissue may impede drug diffusion and drug availability may be limited by interstitium-dwelling cell absorption. Drugs delivered systemically may also have detrimental and off-target effects. Targeted drug delivery attempts to address these concerns by delivering drugs selectively to the designated tissue while limiting exposure to healthy tissues. This method can reduce potential damage while increasing therapeutic efficacy. One example of tailored medication delivery is the use of liposomes as drug carriers. Liposomes, which are spherical vesicles comprised of a lipid bilayer and can protect drugs from degradation, can be used to encase them. Liposomes can be directed to certain tissues or cells by attaching targeting ligands to their surface, such as antibodies or peptides. Liposomes modified with a lymphatic-homing peptide, for example, have been shown to congregate in LNs following subcutaneous injection, improving the delivery of vaccines and cancer medicines to these tissues [30].

In essence, lymph node-specific drug delivery carriers have the potential to improve the effectiveness of drug delivery to these nodes. By using a tailored strategy, the overall amount of medication needed can be decreased, reducing toxicity and off-target effects [30, 31].

### Lymphatic targeted delivery system

The lymphatic system has crucial functions in the body which include maintaining the balance of fluid levels in tissues as well as the transportation of cells involved in immune response leading to their maturity in LNs. However, this system is also involved in tumor spreading to other areas of the body following metastasis [32]. Therefore, the need for such targeting compounds and drug carriers is crucial which can target sites of the lymphatic system and reduce side effects of already available approaches. In a study, researchers successfully synthesized and analyzed DEC205-PAPP nanoparticles. The immunomodulatory effects of DEC205-PAPP as a novel antigen adjuvant delivery method were investigated in a series of tests, and the results revealed that DEC205-PAPP could greatly improve the immune response. DEC205-PAPP increased macrophage uptake activity in vitro. Furthermore, DEC205-PAPP increased macrophage production of M1-type cytokines IFN-, IL-6, and GM-CSF, as well as the expression of M1 polarization indicators (CD80 + and CD86+) [33]. LH-SMEDDS was effectively produced and characterized in a study to improve its low bioavailability. The created LH-SMEDDS microemulsion had a narrow globule size distribution, a spherical shape, excellent transmittance, and a short self-emulsification time. Caco-2 cells showed enhanced intestinal absorption and uptake in cell line tests. LH-SMEDDS successfully increased bioavailability to the same extent as LH suspension in rats via intestinal lymphatic transport. As a result, it can be stated that LH-SMEDDS is capable of increasing LH bioavailability after oral administration by employing bioactive excipients [34]. Lymphatic-targeted drug delivery systems can be achieved through both active and passive targeting.

### Active lymphatic targeting

Active targeting is the use of ligands or antibodies that firmly attach to tumour cell receptors or antigens [35]. When a tumour develops, cancer cells emit a range of vascular endothelial growth factors (VEGFs). These VEGFs can encourage the formation of lymphatic channels inside tumours due to their ability to bind to specific receptors on lymphatic endothelial cells (LECs). As a result, this method facilitates cancer spread via the lymphatic system [36].

Chemical methods were used to produce CaCO_3_ NPs with an average diameter of 58 nm and a zeta potential of + 28.6 mV. The small SiRNA intended to be delivered by these NPs were designed to target VEGF-C. In the SGC-7901 human gastric cancer cell line, siRNAs directed against VEGF-C in calcium carbonate NPs outperformed non-specific siRNAs in terms of transfection efficiency. The levels of VEGF mRNA and VEGF-C were lowered by over 80%, confirming this improvement. Animal investigations revealed that complexes of CaCO_3_ NPs with VEGF-C-targeting siRNAs could effectively suppress lymph angiogenesis, limit regional lymph node metastasis, and prevent primary tumour growth. The test group showed a much-reduced rate of around 20% compared to the control group, which had a tumour lymphatic metastasis rate of 70% [37].

A recent study has led to the identification of the nine-amino acid cyclic peptide LyP-119. Notably, this peptide has a particular affinity for tumour lymphatic cells, tumour cells, and tumor-associated macrophages [38]. A LyP-1-conjugated liposome-based medicine delivery system was developed in another study specifically to target lymphatic metastatic cancers. These liposomes are designed to be injected subcutaneously or intramuscularly before being transported into the lymphatic system. Liposome absorption by tumor-associated metastatic LNs is improved by the LyP-1-mediated targeting mechanism. These liposomes generate anticancer drugs that attack cancer cells while also harming lymphatic conduits within LNs and tumor-associated macrophages. As a result, the inhibitory effect of this LyP-1-conjugated liposome drug delivery method on lymph node metastasis is considerably enhanced [39].

### Passive lymphatic system

Passive targeting is the use of the lymphatic system’s inherent properties to enhance medication delivery. For example, chyle and NPs administered orally are absorbed by lymphatic channels and Pye’s LNs, whereas particles administered subcutaneously are absorbed by lymphatic vessels and LNs. It is possible to selectively target specific body regions with nanoparticles of varying sizes. The determination of whether nanoparticles can get past various barriers to gain entry into LNs is contingent upon the particle size, which is a crucial factor [40]. Small molecules with a molecular weight of less than 20 kilo Daltons (KD) or NPs with a size of less than 10 nanometers are capable of easily crossing the capillary walls and can be promptly eliminated following administration [41]. Molecules with a size greater than 20 KD or NPs ranging from 10 to 100 nanometers in size can undergo passive diffusion across the inter-endothelial junctions and enter the lymphatic vessel [42]. Research has demonstrated that the most effective particle size range for direct transportation to lymph nodes based on size effects is between 20 and 50 nanometers. This finding serves as the foundation for the development of targeted nano-delivery systems intended for LNs [43, 44]. When injected intravenously, macrophages from the reticuloendothelial system (RES) of the liver, spleen, lung, and bone marrow are capable of removing emulsion droplets that are between 0.1 and 0.5 m in size [45]. Fat emulsion droplets do not circulate through the liver. Instead, the vast majority of these droplets in the RES congregate inside macrophages. Because of the superb lymphatic system targeting made possible by this aggregation process, the medicine’s anti-inflammatory properties are improved. Mitomycin C, the first medicine for lymphatic targeting, can be given intravenously or subcutaneously as water in oil (W/O) or oil in water (O/W) emulsions. W/O emulsions, according to previous studies, target the lymphatic system more effectively than O/W emulsions. Furthermore, encapsulating a drug in NPs before dispersing it in oil emulsions can improve the drug’s lymphatic targeting significantly [46].

**Fig. 3:**
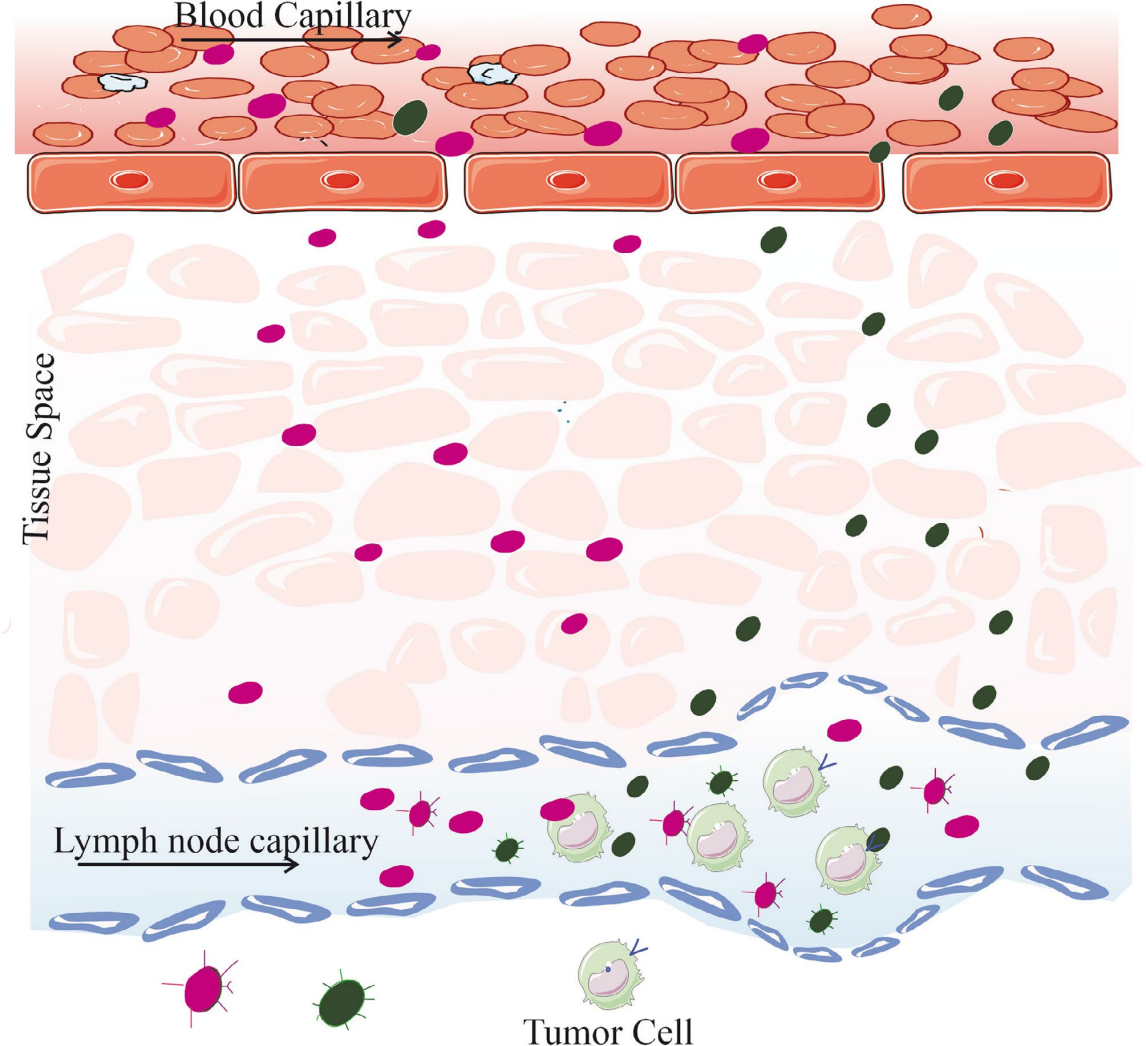
Unleashing the Potential of Nanocarriers. Targeting lymphatic capillaries for improved accumulation in LNs

When capillary lymphatic capillaries are produced by monolayer endothelial cells during the lymph generation process in disorders such as cancer or inflammation, their basement membranes are insufficient and exhibit excessive permeability. The lymphatic drainage channels can also grow to widths of 300–500, although having a normal width of 30–120 nm [47]. Comparatively, blood capillary distances are less than 10 nm. Aside from traveling via the bloodstream, macromolecules, and colloids of the appropriate size (100 nm) can diffuse into the lymphatic drainage system (Fig. 3). It’s important to understand that as these macromolecules or colloids move through the LNs, macrophages may seize them. As a result, this approach can be used to target lymph node metastases passively.

Both active and passive targeting have their advantages and limitations, and further research is needed to optimize their use in lymphatic-targeted drug delivery systems [45, 46]. The afferent lymphatic duct allows particles from various tissues to enter the LNs, which act as filters for bodily fluids [48]. The ability of NPs introduced into the bloodstream to access the lymphatic vessels depends on their size. NPs with diameters that span 10 to 100 nm have lymphatic vascular accessibility, owing to the rapid passage of small molecules from the circulation into the lymphatic system [31]. Conversely, larger particles tend to remain in the interstitial matrix and face challenges in accessing the lymphatic fluid. The size varies from 10 to 100 nm, smaller NPs have a tendency to accumulate in metastatic LNs, suggesting a preferential migration to these LNs. This phenomenon can be attributed to the unique properties of smaller NPs that facilitate their migration to lymphatic vessels and subsequent accumulation in metastatic LNs.

Particle properties other than size play a crucial role in lymphatic delivery [49]. Flexible NPs have shown advantages over rigid ones in penetrating the interstitial space and accessing lymphatic vessels, while particles with negative surface charges exhibit enhanced migration in the interstitial matrix. It has also been reported that coating carrier NPs with polyethylene glycol (PEG) also enhances the biocompatibility of nanocarriers to transport the drugs and therapeutic agents to lymphatic systems. Pegylated NPs of sizes 40 and 100 nm showed an increased transport to the lymphatic system by about 50 folds as compared to NPs that were not modified with PEG. This increase in lymphatic transport of nanocarriers was irrespective of the sizes of NPs [50]. Understanding and leveraging these particle characteristics are vital for optimizing targeted drug delivery strategies to the lymphatic system.

### Nanocarriers for targeted drug delivery to lymph node

The lymphatic system is the primary location of metastasis for the majority of solid tumours, and any lesions discovered signal pathological alterations connected to many disorders. However, due to the unique architectural features of the lymphatic system, including metastases in the LNs, it is often difficult for medications to reach their intended target in the lymphatic tissues [51]. Therefore, to improve the therapeutic efficacy of drugs, it is crucial to develop a reliable lymphatic targeted drug delivery system that can effectively transport the drugs to the lesions [52].

Just as NPs have been employed for targeted delivery in a variety of diseases, nanocarriers were investigated as the first carrier system for targeted drug delivery to the lymphatic system. Nanocarriers can be easily manipulated to enhance their site-specific lymphatic targeting and reduce other off-site harmful effects. A wide variety of nanocarriers can be used for targeting lymphatic system and LNs depending on the required outcomes. For delivering drugs and therapeutics agents to LNs by using nanocarriers, both organic and inorganic NPs can be used. It is important to know the site of the target and possible side effects which need to be addressed when selecting the particular type of nanoparticle for lymphatic system drug delivery. Phospholipid vesicles and liposomes are some lipid NPs that have a hollow core and can be used for the encapsulation of drugs for delivery to LNs [53]. By modifying these nanocarriers with neutrally charged coating agents such as PEG, the biocompatibility of these nanocarriers can be enhanced greatly. Also, for easier and site-specific uptake of these nanocarriers, ligands can be attached to the surface of these nanocarriers, and these ligands are specific for endothelial cells present in the lymphatic system, resulting in uptake by LNs. Liposomes also ensure a slow release of the drug. Actinomycin D is an anticancer drug whose uptake by the LNs has been enhanced by encapsulation via liposomes [54].

LNs serve as the primary location for immune cells, including antigen-presenting cells (APCs), T cells, and B cells. These cells are enveloped by a delicate fibrous capsule and are capable of detecting antigen signals via the afferent lymphatics. Beneath the capsule, there exist barrier cells, including macrophages and other types, which are formed within the subcapsular sinus (SCS). Research findings indicate that the depletion of SCS macrophages results in increased accessibility of LNs to nanoparticles. The cortex, situated below the subcortical structures (SCS), comprises small channels measuring 3–5 nanometers in width [55, 56]. Within the LNs, the cortical and medulla areas are primarily separated, with the capsule connective tissue spreading towards the parenchyma to produce trabeculae. The cortex and paracortex are structurally distinct cortical sections. The cortex is the primary location of B lymphocytes and spherical follicles. The initial follicles are not activated by antigens, and the spherical follicles lack germinal centers. Follicular dendritic cells (FDCs) can collect antigens after antigen stimulation and cross-link B cells for antigen delivery. When introduced nanocarriers encounter subcapsular sinus as they enter lymph nodes. The size and charge present on the surface of the nanocarrier can greatly influence the interaction with the subcapsular sinus. The subcapsular sinus which is situated under the LN capsule, acts as an initial filter for nanocarriers, where they come in contact with macrophages and other cells. Then in the paracortical region (T-cell zone), rich in endothelial venules allows lymphocytes to enter. Nanocarriers can concentrate here because of their size, surface charge and unique receptor interaction. In medullary cords, plasma cells, B-cells and macrophages are present in the inner lymph node regions. Nanocarriers can reside in these cords based on their surface characteristics. The distribution of nanocarriers in different regions of lymph nodes can greatly influence targeted drug delivery and diagnostic applications.

### Importance of physicochemical parameters of nanoparticles

The interaction of nanoparticles with biological systems is primarily influenced by several key characteristics, namely their size, shape, hydrophilicity/hydrophobicity properties, surface coating composition, the presence of functional groups, and their charge. The diminutive dimensions of nanoparticles are a primary attribute that facilitates their ability to traverse biological barriers, penetrate cells, and translocate throughout various cells, tissues, and organs [57]. The toxicity of nanoparticles has been observed to increase with decreasing size.

In addition to nanoparticle size, the shape of nanoparticles is also recognized as a significant determinant of their functional properties. Nanoparticles have the capacity to be synthesized in a multitude of shapes, including but not limited to tubes, fibers, and spheres. Research indicates that the morphology of nanoparticles is a crucial factor in cellular internalization, circulation within the bloodstream, anti-tumor efficacy, and biodistribution [58, 59]. The interactions between cellular compartments and nanoparticles are significantly influenced by the surface properties of NPs. The surface charge and density of nanoparticles have been identified as properties that can influence the extent of cellular uptake and the interaction of particles with biomolecules [60].

### Liposomes based Nano drug delivery system

Bangham and his colleagues discovered liposomes, also known as phospholipid vesicles, in the 1960s. These hollow vesicles are lipid NPs comprised of lipid bilayers. A variety of drugs can be encapsulated within the lipid bilayers or their hollow structures. Even if they are biocompatible, medicinal drugs must be given particularly to LNs [47]. Some of the parameters that must be carefully managed are size, charge, polyethylene glycol (PEG) modification, and the insertion of ligands that target APCs [61]. The use of LN-targeting liposomes is a potential technique for effective immune activation in vaccine delivery and anti-tumor therapy [62].

The majority of subunit vaccines require the use of adjuvants to improve antigen absorption and produce measurable immune responses with little damage [63]. Liposomes have become a popular vaccine adjuvant due to their ability to securely encapsulate and distribute a wide spectrum of compounds in vivo [64]. Liposomes, in addition to functioning as adjuvants for protein vaccines, can protect nucleic acid vaccines against enzymatic degradation and facilitate their entry into the cytoplasm. Numerous trials have demonstrated the potent anti-tumor effectiveness of liposome-based nucleic acid vaccines. Maeta’s research team, for example, developed liposome NPs for use as in vivo DNA vaccines. These liposomes include a lipoid molecule (SsPalm) that responds to pH variations by activating. Various ssPalms with distinct hydrophobic scaffolds have been studied, and the ssPalmE type, which incorporates α-tocopherol as the scaffold, demonstrated notable efficacy in delivering nucleic acids (siRNA/pDNA) to the liver following intravenous administration. The material contains two functional units that allow modulation of intracellular trafficking; tertiary amines for pH-responsive membrane instability and a disulfide bond for cytoplasmic collapse [65]. The initial SsPalm molecule was produced using hydrophobic scaffolds in the form of saturated fatty acids, specifically myristic acid (ssPalmM). The LNP complex comprising of the ssPalm (LNPssPalm) exhibited a greater efficacy in pDNA transfection as compared to the LNPs containing the conventional ionizable cationic lipid DODAP and the non-cleavable counterpart of SsPalm, ccPalm, wherein the disulfide bond was substituted with a -CH2-CH2- bond [66]. In the draining LNs, dendritic cells (DCs) and macrophages, particularly medullary macrophages, take them up. This liposome formulation’s increased gene expression activity results in significant anti-tumor or antiprotozoal actions. As a result, it has promise as a DNA immunization against protozoa and cancers [67].

Modified liposomes have been employed in various studies that have revealed unique characteristics in the detection and management of lymph node (LN) problems. Akita’s research team discovered a novel granular formulation containing PS that can be efficiently delivered to sentinel LNs. Liposomes containing PS show effective accumulation and retention in sentinel LNs after HAase binding. As a probe for the focused detection of sentinel LNs, this mixture has a lot of potentially aggregation may be facilitated by CD169-positive macrophages’ internalization of PS-containing liposomes. Furthermore, PS liposomes outperform the currently available imaging agent indocyanine green for sentinel LN imaging. Because of the crucial role that macrophage accumulation plays in the considerable accumulation of LNs, these particles may perform effectively as carriers for antigen or adjuvant injection in cancer immunotherapy [68].

### Nano drug delivery platform based on micelles

Spherical, multilayer, and rod-like micelles are examples of micelles, which are ordered molecular aggregates [69]. Amphiphilic molecules with both hydrophilic and hydrophobic areas make up their structure, allowing them to encapsulate hydrophobic drugs within their core [70]. The interior of the micelle is composed of hydrophobic surfactant molecule segments, and the exterior layer is composed of hydrophilic polar groups [71]. Micelles are widely used to deliver therapeutic chemicals with low water solubility, increasing their solubility and lengthening the blood half-life of chemotherapeutic drugs. Antineoplastic drugs may occasionally passively gather at the cancer site due to the leaky vasculature, enhancing drug permeability and retention [72, 73].

Micelles used in the drug delivery system are designed to be safe and effective at delivering medication. A polymer micelle constructed of methyl polyethylene glycol distearyl phosphatidylethanolamine (mPEG-DSPE) increased adriamycin’s absorption in A375 cells. Liposomes are often prepared using methoxy-polyethyleneglycol-distearyl phosphatidyl-ethanolamine (mPEG-DSPE). Its utility in the production of micelles for the transport of poorly soluble pharmaceuticals has also been demonstrated, and as a result, it has attracted a lot of recent attention [74]. A study revealed that mPEG-DSPE micelles could load probucol with excellent efficiency. The Caco-2 cell model was utilized for in vitro assessment, revealing that the micellar formulation exhibited a noteworthy improvement in the absorption and transportation of probucol. The research conducted on rat models with cannulated lymph ducts, both conscious and unconscious, provided additional evidence supporting the notable enhancement of lymphatic transport of mPEG-DSPE micelles [75]. Subcutaneously injected micelles target and destroy cancer cells within LNs by being absorbed by the LNs and accumulating in the draining LNs. Despite the fact that Adriamycin can cause tissue damage, Micelles produce less tissue damage than Adriamycin alone [76]. Methoxy polyethylene glycol-b-polylactic acid (mPEG-PLA) and mixed poly (Dmurl-lactic acid-glycolic acid) (PLGA/mPEG-PLA) are another type of nanoparticle (NP) created for lymphatic uptake. The small chemical resquimod, which functions as a TLR7 agonist, can be delivered via these particles. Numerous studies have demonstrated that when taken up by DCs and macrophages, PLGA/mPEG-PLA particles loaded with resquimod can trigger an anti-tumor immune response. Resquimod is systemically harmful when delivered by other methods. Subcutaneous treatment, on the other hand, exclusively promotes cytotoxicity in tumour cells while causing no harm to immune cells [77].

The targeted administration of protein antigens to LNs via micelle binding has been shown to successfully increase the cellular immune response following cutaneous administration. This method significantly improves CD8 + T cell memory capacity as well as the cellular immunological activity of antigen-specific CD8 + and CD4 + T cells [78, 79]. Micelles have been found to accumulate in LNs and exhibit features that inhibit cancer lymphatic dissemination. Dendritic cell (DC) activation and CD8 + T cell cytotoxicity are both linked to LN tumour growth inhibition [80]. Small micelles (less than 50 nm in size) have shown promise as a conservative therapy for suppressing lymph node metastases, resulting in decreased recurrence rates and better survival outcomes. New non-invasive sarcoidosis therapeutic options are now available, thanks to the utilization of elastic therapy and the selective aggregation of nano-micelles in metastatic LNs. Reddy et al., for example, used PPS NPs stabilized by Pluronic’s as a platform for antigen transfer. These ultra-thin NPs (25 nm) effectively reach lymphatic capillaries and drain LNs via interstitial flow after intradermal injection, concentrating on DCs in half of the LNs. The surface chemistry of these NP successfully activates DCs, complement cascades, and local danger signals [81].

### Polymer based targeting delivery system

Polymeric NPs and polymer-modified micelles can be used for lymphatic system drug delivery. As compared to liposomes, these nanocarriers can exhibit versatile surface modifications due to polymer attachment. By conjugation of polymeric nanocarriers with lymph node specific ligands, antibodies, or peptides, they can be targeted for drug delivery to LNs. Poly(lactide-glycolide) micelles encapsulation toll-like receptor-3 ligand poly has been observed to enhance the residence time of ligands and activation of DCs in LNs [82].

### Inorganic NPs Delivery Systems

Another class of nanocarriers involves inorganic NP including gold nanocarriers, mesoporous silica NPs, iron oxide NPs and quantum dots etc. One major advantage of metallic and other inorganic nanocarriers is their ability to be used in combination with other therapeutic approaches such as photo-thermal therapy (PTT). Nanocarriers with magnetic and electric properties have been conjugated with anti-cancer drugs and observed for their effects on the target efficiency of the drug and drug release. Such NPs can be used for drug delivery to lymphatic system due to an enhanced retention time of NPs in lymphatic system and can be imaged through magnetic resonance imaging (MRI) or by tagging with fluorescent antibodies for increased site-specific targeting [83].

Inorganic nano-carriers are used to transport therapeutic compounds to specific cancer sites, primarily via afferent lymphatic channels. The structure of LNs is altered by tumour, both primary and metastatic, boosting fluid and molecular diffusion and enabling drug carriers to enter these LNs more deeply than they would in healthy LNs. Combining photothermotherapy (PDT) with NPs collected in LN cancers allows heat-induced drug activation to exert anti-tumor effects while reducing negative effects [31, 84].

### A new class of highly effective drug delivery nanocapsules

Much research has been done on nanocapsules as pharmaceutical delivery vehicles, with a focus on the lymphatic system. By carefully regulating critical variables such as size, dispersion, biocompatibility, and stimuli-response, nanocapsules have demonstrated their promise for successfully delivering drugs to the lymphatic system. These results demonstrate the value of optimism in these variables in order to optimize the exact delivery of medical medications to lymphatic tissues [70, 85].

The biological distribution and duration of therapeutic efficacy are significantly influenced by the size of nanocapsules. According to Vicente’s study team, smaller polyamino acid nanocapsules (100 nm) demonstrated higher biodistribution and faster access to lymphatic arteries than bigger nanocapsules (200 nm). Docetaxel loading and sustained release were also demonstrated using the 100 nm nanocapsules. Another type of nanocapsule, comprised of polysaccharide shells, progressively migrated away from the injection site and accumulated in the LNs that drain the area. These nanocapsules formed a reservoir at the injection site, allowing for delayed lymphatic drainage and prolonged retention in the lymphatic system [86].

Customized nanocapsules are ideal for loading antigens and adjuvants because they have desirable qualities such as a narrow particle size distribution and high biocompatibility. According to Li and colleagues’ findings, when APCs are encapsulated in lipid nanocapsules containing protein or peptide antigens, they are more easily taken up and delivered to draining LNs. When compared to soluble antigens and adjuvants, the combination of these antigen-loaded nanocapsules and Toll-like receptor agonists revealed better therapeutic efficacy for cancer vaccines and preventive viral vaccines [87].

Furthermore, nanocapsules may improve the oral bioavailability of drugs that aren’t easily dissolved. Attili-Qadri and colleagues demonstrated that lymphatic transport increases the oral bioavailability of docetaxel. Oral nanocapsules containing docetaxel and coated with apolipoprotein and phospholipids have the potential to pass intestinal cells and reach intestinal lymphatic capillaries. Because of this method, docetaxel is exposed for a much longer amount of time, increasing its oral bioavailability [88].

### Nano platforms for diagnosis of lymph node metastasis

Nanocarriers have been extensively studied for targeted drug delivery, drug retention analysis, and pharmacokinetics. One of the main advantages of using nanocarriers to deliver drugs to LNs is that nanocarriers can cross membranes and blood brain barriers to enter the lymph system of the body [89]. Another advantage associated with nanocarriers is the physicochemical nature of nanocarriers. Nanocarriers have a larger surface-to-volume ratio, which is especially beneficial when the main objective is to absorb large doses of drugs into a delivery vehicle. Similarly, nanocarrier surface properties can be modified by binding certain ligands, antibodies, polymers and biological molecules. By functionalizing the surface of nanocarriers with a specific target ligand, such as lymph node epithelial cells, it improve the efficiency of specific targeting areas and reduce drug transport outside the target and thus the associated side effects [2]. The drugs encapsulated by nanocarriers also increase the immune system as a target of the natural body-cleaning mechanism and can stay in circulation for longer periods, thus increasing the drug’s half-life. When selecting a specific nanocarrier for the desired purpose, it is necessary to assess the biodegradability and biocompatibility of the nanocarrier. The nanocarrier must be highly biocompatible and not activate the immune response in order to provide safe and targeted drugs to the required location. Another aspect to consider is the stability of the nanocarriers used. However, the challenges associated with the use of nanocarriers in cancer therapy and diagnosis are not significant, and the advantages of using nanocarriers in the lymphatic system outweigh the current challenges. More evaluation is needed to determine how to increase the efficiency of drug delivery using nanocarriers by increasing stability, reducing the toxic effects of nanocarriers, and using nanocarriers for combined therapy.

Nanosystems have been developed for the diagnosis of lymphatic system metastasis because of the unique optical and physicochemical properties of nanoparticles. For imaging of metastasis in LNs, those NPs should be selected which have certain properties. NPs that are metallic in nature and have magnetoelectric properties are preferred for imaging applications using techniques such as MRI.

Ferumoxtran-10 is a dextran-coated paramagnetic iron oxide NPs that are used as contrast agent in MRI. The clinical trial ACRIN 6671/GOG 0233 evaluated the use of ferumoxtran-10 MRI in detecting lymph node metastasis in advanced cervical cancer. This trial involved multiple medical institutions across various countries and aimed to determine whether the utilization of ferumoxtran-10 could enhance the accuracy of MRI in detecting lymph node metastasis. The findings demonstrated that ferumoxtran-10 MRI exhibited superior sensitivity and specificity compared to conventional MRI techniques. These results suggest that ferumoxtran-10 holds promise as a potential nanocarrier for diagnosing lymph node metastasis not only in cervical cancer but also in other types of malignancies [90].

NPs which are smaller in size and thus are able to effectively cross basement membranes and capillary walls offer better choices as contrast agents. Another important aspect of using NPs in imaging is the ability to modify surface of NPs using functional groups, biological molecules, ligands, and moieties to target specific cells (such as LNs specific cells) and greatly increase the effectiveness of metastasis imaging in LNs. Studies have investigated imaging nanomaterials containing pH-responsive polymers for metastatic sentinel LNs (SLNs) [91]. These nanomaterials utilize self-assembling micelles connected to luminol and a fluorescent probe, which switch on their pH-sensitive imaging capability. In an acidic environment, such as phagosomes within inflammatory macrophages, the NPs disassemble and regain their luminescence. Another study demonstrated the detection of metastatic LNs using pH-sensitive NPs that undergo structural changes in response to proton concentration [92]. These NPs show fluorescence amplification in metastatic LNs, as they disassemble due to cancer acidosis. Enhancing nanoparticle delivery to metastatic LNs was achieved by degrading hyaluronic acid, increasing the permeability and retention effect [93]. The combined treatment of hyaluronidase and nano micelles effectively inhibited primary tumor growth and promoted the accumulation of nano micelles in metastatic LNs. pH-responsive polymers and systems are sensitive to cancer acidosis offer indirect methods for detecting metastatic SLNs, complementing other probes and signaling pathways. Mannan-functionalized NPs target mannose receptors on APCs [94, 95]. They have been applied to MR imaging of LNs, where the NPs are mostly taken up by antigen-presenting cells, activated macrophages, and DCs [95]. Mannan-capped gold NPs also enhance X-ray contrast for imaging of popliteal LNs [94]. The targeting mechanism involves mannose receptor-mediated endocytosis. These findings highlight the potential of mannan-functionalized NPs for targeted lymph node imaging. Iron oxide NPs have been studied for imaging of LNs metastasis by delivering NPs through intravenous injection. These NPs are about 30 to 1000 nm in size and can cross the membranes of capillary, accumulating in LNs after intravenous injection. Later, routine procedure of magnetic resonance imaging can be applied to detect the presence of these NPs in LNs acting as a contrast agent [96]. SLNs have been imaged through a polyester coated nanoparticle made up of gadolinium oxide and coated with cyclodextrin for metastasis detection. Some NPs accumulated in SLNs, but the efficiency of detection was lower as compared to the available contrasting agents [97]. Patients in earlier stages of breast cancer have also been imaged using carbon NPs for detecting SLNs. After introduction of carbon NPs into the SLNs, SLNs were stained black due to the characteristic color of carbon nanoparticles. As a result, about all the patients were successfully imaged for SLNs after biopsy was analyzed [98]. This method of imaging SLNs was found to be efficient, and it is reported to be easier than the conventional blue dye method used for imaging SLNs.

### The effect of NP size on lymphatic targeting

The distribution and interactions of NPs with immune cells after delivery are heavily controlled by their size. To control the size of polymeric and lipid NPs, one can change the physicochemical properties of the substances used for NP synthesis or use various NP preparation techniques such as flash nanoprecipitation, microfluidic devices, layer-by-layer self-assembly, and particle replication in nonwetting templates (PRINT) technology. These strategies have been extensively discussed in the scholarly literature [99].

Draining LNs are a prime choice for vaccines and therapies because they contain a large number of immature APCs [100]. The size of the NPs has been identified as an important factor in LN targeting and retention. Smaller PPS NPs(50 nm) stabilized with PEG or pluronic were more effectively transported to draining LNs and processed by immature DCs after intradermal (i.d.) administration than larger NPs (100 nm) via convective lymphatic drainage [101]. Using dendritic polymer, the lowest size limit for preferred lymphatic drainage targeting was discovered to be 8 nm [102].

The interstitial extracellular matrix, which functions as a molecular filter for convective transport with holes of around 100 nm in diameter, has an impact on size discrimination in lymph node (LN) targeting [103]. DCs move to the draining LN and pick up NPs larger than 100 nm, which are maintained at the injection site [104]. This size restriction can be overcome by intra-LN injection [82]. Low-molecular-weight compounds can pass through the CD169 + subcapsular macrophage lining and enter the paracortical zone, which has dense concentrations of T cells and DCs [105]. Unless coated by complement, larger NPs stay in the subcapsular sinus and eventually reach the bloodstream without being taken up by immune cells [106].

### NP shape’s impact on lymphatic targeting

Researchers may now tailor the shape of these particles because of advances in NP preparation techniques such as PRINT, step and flash imprint lithography (S-FIL), and membrane stretching [107, 108]. This has opened up new avenues for research into how the NP form affects various applications. NPs can be detected and taken up by macrophages through the process of phagocytosis regardless of how they are consumed. This approach may have a huge impact on the delivery effectiveness of non-targeting macrophage NPs to the selected cell groups. It has long been recognized that the NP shape influences how efficiently macrophages engage in phagocytosis. In mice and rats, for example, worm-like filomicelles that administered the anti-cancer medication paclitaxel had a blood circulation period ten times longer than their spherical counterparts. Filomicelles were found to be more challenging to macrophage phagocytosis than spherical micelles in both cell culture and flow settings [109]. Table 1 below summarizes some studies assessed for the treatment of various ailments using nanocarriers in the recent past.

**Table 1:** Nanocarriers used for the treatment of various diseases in recent era

Nanoparticles	Advantages of Drug delivery approach	Disease	Reference
Nano-diamonds	The transport of small interfering RNA into sarcoma (Ewing) cells can be facilitated by nano diamonds.	Cancer	[110]
AgNPs	The process included developing a stable silver NP vector in order to create mosquito larvicides, which are medications that are used to kill mosquitos.	Dengue fever or malaria	[111]
Polyamidoamine nanoparticles	Polyamidoamine nanoparticles act as nanocarriers, delivering anti-malarial drugs to specific areas.	Malaria	[112]
Solid Lipid NPs	Electroporation and nanocarriers are utilized for drug delivery.	Colon cancer	[113]
Mesoporous silica NPs	This method is a pH-sensitive drug delivery system constructed from folic acid-targeted HBP reforming MSNPs.	Cancer	[114]
Lapatinib-loaded human serum albumin	Restriction of 4T1 cell motility, invasion, and adhesion in the brain.	Brain metastasis	[115]
Au-Fe nanocomposite	Cytotoxicity against malignant U87MG cells was higher than against normal astrocyte cells.	Brain cancer	[116]
Chitosan NPs	NPs exhibit cytotoxicity when used against C6 cell line and can inhibit MDCKII-MDR1 cell growth.	C6 glioma cells	[117]
Lipid–drug-conjugated NPs	When 5-FU was synthesized with LDC nanoparticles, its efficacy in treating brain cancer was increased.	Brain cancer	[118]

## Conclusion

LNs metastasis is a significant factor in cancer progression, with 90% or more cancer cells passing through lymph nodes in the lymphatic system. The prognosis of malignant cancers is heavily influenced by lymph node metastasis, making research on sentinel lymph node mapping popular. To develop effective treatments and better prognostic options, it is crucial to analyze lymph node metastasis and develop targeted techniques and agents. Regularly imaging LNs to detect resident cancer cells is essential to prevent relapse in cancer patients. Understanding the metastatic profile of LNs is crucial for selecting the right chemotherapeutic agent and minimizing treatment side effects. Nanocarriers and NPs have been used for disease diagnosis and therapeutic strategies in many diseases, including cancer. Nanocarriers can be coated with polymers and moieties to hide them from immune responses and can be functionalized with antibodies, chemical agents, and ligands. They can also enhance the efficiency of conventional imaging techniques like MRI by utilizing optical and electromagnetic properties of NPs like gold and iron oxide NPs. These nanosized carriers can be precisely controlled to achieve a specific size, increasing drug loading efficiency and reducing off-target side effects.

In recent years, nanomedicines have been introduced into the clinical practice with approval from regulatory agencies. Many clinical trials on nanomedicines are underway for their successful translation to clinics. A clinical trinal on dextran-coated paramagnetic iron oxide NPs (Ferumoxtran-10) has been conducted for detecting lymph node metastasis in advanced cervical cancer. The findings of this trial demonstrated that ferumoxtran-10 exhibited superior sensitivity and specificity compared to conventional MRI techniques. Though, the results of many in-vitro and in-vivo studies are promising, regulatory approval of nanomedicines is challenging as detailed particle characteristics influencing its kinetics and ultimately the toxicity, biocompatibility, stability, uniformity, bio-distribution and intracellular fate of the product need to be specifically elucidated. Thus, extensive research is needed to address the challenges and side-effects of these carriers, as they can be efficient means of both imaging and targeted drug delivery to LNs.

## Data Availability

All data regarding this manuscript is available in the main text.
